# Contextual and individual factors associated with dental services utilisation by Brazilian adults: A multilevel analysis

**DOI:** 10.1371/journal.pone.0192771

**Published:** 2018-02-08

**Authors:** Fernando José Herkrath, Mario Vianna Vettore, Guilherme Loureiro Werneck

**Affiliations:** 1 Escola Superior de Ciências da Saúde, Universidade do Estado do Amazonas, Manaus, Amazonas, Brazil; 2 Instituto de Medicina Social, Universidade do Estado do Rio de Janeiro, Rio de Janeiro, Rio de Janeiro, Brazil; 3 Instituto Leônidas e Maria Deane, Fundação Oswaldo Cruz, Manaus, Amazonas, Brazil; 4 Academic Unit of Oral Health, Dentistry and Society, School of Clinical Dentistry, University of Sheffield, Sheffield, United Kingdom; 5 Instituto de Estudos em Saúde Coletiva, Universidade Federal do Rio de Janeiro, Rio de Janeiro, Rio de Janeiro, Brazil; University of Washington, UNITED STATES

## Abstract

**Background:**

Inequalities in the utilisation of dental services in Brazil are remarkable. The aim of this study was to evaluate the association of contextual and individual factors with the utilisation of dental services by Brazilian adults using the Andersen’s behavioural model.

**Methods:**

Individual-level data from 27,017 adults residents in the State capitals who were interviewed in the 2013 Brazilian National Health Survey were pooled with contextual city-level data. The outcomes were non-utilisation of dental services and last dental visit over 12 months ago. Individual predisposing variables were age, sex, race/skin colour, schooling and social network. Individual enabling variables included income, health insurance and registration in primary health care. Individual need variables were self-perceived dental health and self-reported missing teeth. Multilevel logistic regression models were performed to estimate odds ratio (OR) and 95% confidence intervals (95% CIs) of the association of contextual and individual predisposing, enabling and need-related variables with dental services outcomes.

**Results:**

Predisposing (OR = 0.89; 95% CI 0.81–0.97) and enabling (OR = 0.90; 95% CI 0.85–0.96) contextual factors were associated with non-utilisation of dental services. Individual predisposing (sex, race/skin colour, schooling), enabling (income, health insurance) and need (self-perceived oral health, missing teeth) were associated with non-utilisation of dental services and last dental visit over 12 months ago. The latter was also associated with other individual predisposing (age, social network) and need (eating difficulties due to oral problems) characteristics.

**Conclusions:**

Individual and contextual determinants influenced dental services utilisation in Brazilian adults. These factors should be on the policy agenda and considered in the organisation of health services aiming to reduce oral health inequalities related to access and utilisation of dental services.

## Introduction

Public oral health care in Brazil has been historically restricted to a limited range of dental procedures offered in the large urban centres. In this context, curative dental procedures have been predominating over preventive dental services and access to oral health care has been restricted to few population groups [1]. However, over the recent years, the organisation of public dental services has been improved with the implementation of the National Oral Health Policy. The core principles of the policy focus on the reorganisation and expansion of primary oral health care within the Brazilian Unified Health Care System (SUS). In addition, the policy framework proposes the increase in the provision of universal dental care through combining actions at individual and collective levels encompassing health promotion, prevention, diagnosis, treatment and rehabilitation [2].

Since 1998 primary care in the SUS has been reorganised around a priority strategy which provides community-based primary health care through Family Health Teams (FHT), that are responsible for the health of the population at a defined geographical area [3, 4]. The gradual incorporation of oral health professionals in the teams occurred since 2000. The implementation of the National Oral Health Policy was accompanied by a significant increase in the federal public funding, resulting in the expansion of access and enhancing comprehensiveness of health care [1, 5–7]. This propitiated the expansion of oral health teams in primary care and the implementation of dental specialties centers for secondary care, offering procedures that were previously exclusive to private services [1, 5].

However, despite the increase in access, large inequalities in dental health care utilisation and in the oral health status of the Brazilian population remain noticeable. The most vulnerable groups, such as rural, elderly, deprived and less educated populations, face more barriers in using health services and present have worse oral health conditions. Furthermore, epidemiological surveys also revealed marked oral health inequalities between the regions of the country [6–16].

Utilisation of health services results from a complex interaction between users, availability and access of services and is influenced by several factors, including individual characteristics (e.g. perception of health status and health needs), contextual factors (e.g. social inequalities) and organisation of health services (e.g. distribution of health care units) [17–19]. Previous studies on the associated factors highlight that dental services utilisation results from the interaction between biological, sociocultural, family and community determinants, as well as from the characteristics of health systems [20, 21]. The literature also suggests that reducing social inequalities plays an important role in mitigating barriers to health services utilisation [7, 22–24].

Dental care utilisation and frequency of dental services utilisation by Brazilian adults have been associated with high schooling [7, 8, 10, 12, 22, 25–32] and better-off socioeconomic status [8, 12, 26–29, 32]. Females [7, 8, 26–30], younger people [7, 8, 22, 26, 28, 33], white race/skin colour [7, 34] and having a health insurance [7, 27, 30] have also been associated with a greater utilisation of dental care. Regular users have less dental treatment needs and better oral health conditions [12, 22, 25, 26, 29, 31], and better general and oral health self-perception [26, 28, 29, 31, 32]. Contextual factors, such as living in urban areas [8, 33] and in locations covered by the FHT in primary care, have been linked with an increased likelihood of health services utilisation [7].

Similar findings regarding inequities in the oral health conditions and utilisation of services have been reported in other countries. Populations and countries with greater social inequalities present higher burden of oral diseases and lower use of dental services [20, 23, 24, 35–42]. Investigating the associated factors of utilisation of dental services is relevant for planning realistic interventions to improve access and quality of health care, reorganisation of health services, and ultimately to improve the use of available resources [21, 43]. The identification of the determinants of non-utilisation dental services as a way of assessing barriers to access to dental services can contribute to the improvement of equity in oral health care, but recent data from Brazil have not yet been extensively explored for this purpose. Thus, the aim of the study was to evaluate the association of contextual and individual factors with the utilisation of dental services by Brazilian adults living in the State capitals.

## Methods

### Study design

This cross-sectional study used secondary data from the Brazilian National Health Survey (NHS), a nationwide home-based survey conducted in 2013 by the Ministry of Health in partnership with the Brazilian Institute of Geography and Statistics (IBGE). The purpose of NHS was to evaluate the health status and lifestyles of the Brazilian population, as well as health care, including utilisation and access to health services, continuity of care and financing [44]. All data are fully available without restriction available through IBGE homepage (https://ww2.ibge.gov.br/english) and database (ftp://ftp.ibge.gov.br/PNS/2013/microdados/pns_2013_microdados_2017_03_23.zip).

### Study population

The NHS sample was representative for Brazil, geopolitical macro-regions, States, metropolitan regions and States capitals. Census tracts or a set of these with at least 60 households was defined as the primary sampling unity (PSU). Sampling was performed in three stages. In the first stage, census tracts were selected with probability proportional to size (given by the number of households in each unit) in each stratum. A simple random sampling of households was selected in the second stage in each PSU selected in the first stage. The third stage was a simple random sampling of one adult aged 18 years or older among all adults living in the household to answer a comprehensive questionnaire. The present study analysed data from 27,017 adult dwellers in the 27 State capitals.

### Study variables

The two outcome variables related to utilisation of dental services were based on the following question: “When was your last dental appointment?”, with the following response options: 1. In the last 12 months; 2. From one year to less than two years; 3. From two years to less than three years; 4. Three years or more; 5. Never had a dental appointment. The categories were grouped in order to derive two outcomes: (i) non-utilisation of dental care, considering the option “never had a dental appointment” as outcome (response option 5 vs 1–4); and (ii) last dental visit over 12 months ago, assessed only among those who have already been to the dentist (response options 2–4 vs 1, those who answered option “5” were excluded from this analysis).

The selection of independent variables was based on Andersen’s Behavioural Model of Health Services Utilisation [43]. Individual predisposing characteristics included age, sex, self-reported race/skin colour, according to the categories used in Brazil by the IBGE (white, black, yellow/Asiatic, brown/“pardo” and indigenous) [45], schooling (years of study completed with approval) and social network. Social network was assessed using the items proposed by Berkman and Syme [46], categorising individuals into four groups: I, being the more isolated category, and II, III or IV composing the other three categories of gradation in social relations. Individual monthly income, health insurance and registration in primary care FHT were selected as enabling factors. Perceived need included self-perception of oral health and self-reported difficulty to eat due to oral problems and evaluated need was assessed through self-reported number of missing teeth.

Contextual variables included city-level predisposing, enabling and need indicators. Predisposing contextual characteristics included information about social vulnerability for the year 2010, including data on income, education and life expectancy from the Human Development Index obtained from the Atlas of Human Development in Brazil, besides proportion of individuals living in extreme poverty (household *per capita* monthly income ≤ R$70.00) and proportion of individuals vulnerable to poverty (household *per capita* monthly income ≤ R$255.00) [47]. Enabling factors were *per capita* expenditure in primary health care, *per capita* expenditure in oral health programs, and coverage of oral health teams in primary health care obtained from health information systems for the year 2013 [48]. The contextual need variables were number of permanent decayed, missing and filled teeth (DMFT index), self-reported dental pain in the last six months, need for dentures and impact of oral conditions on daily activities (OIDP questionnaire) among adults who participated at the 2010 Brazilian oral health survey. This information were collected at individual level and then aggregated at the city level [49].

### Data analysis

First, the independent variables were described according to the dental services outcomes using proportions and 95% confidence intervals (95% CI), considering the complex sampling design and the sample weights (svyset UPA_PNS [pw = v0029], strata(v0024) poststrata(v00293) postweight(v00292)).

Second, factor analysis was used considering that contextual variables at city-level (n = 27 State capitals) are correlated and represent underlying dimensions related to utilisation of dental services. The oblique rotation method was used considering the existence of a correlation between the contextual variables. The predicted scores of the factors were used in subsequent analyses.

Third, multilevel logistic regression analysis with robust variance was performed to estimate the odds ratios (OR) and 95% CIs of contextual and individual independent variables (fixed effects with random intercept) and the two outcomes: (i) non-utilisation of dental services and (ii) last dental visit over 12 months ago. Logistic regression models were used since they provide more valid estimates of the incidence density ratio when cross-sectional data is analysed [50]. Initially, associations between independent variables and the two outcomes were tested in bivariate analysis. Variables that presented with p<0.10 in bivariate analysis were considered for the multivariate hierarchical multilevel analysis. Stepforward non-automatic selection of variables in four models was conducted to obtain a parsimonious model according to Andersen’s theoretical model. The first model included only the contextual factors. The second and third models consisted of individual predisposing and enabling variables, respectively. The final model included individual need-related variables. Variable that remained statistically significant at 10% (p<0.10) were retained in the analysis for adjustment in the next model. The percentage of the variance explained by the contextual level (variance partition coefficient) was calculated using the standard logistic distribution variance, π^2^/3≅3.29, as the level 1 variance [51]. All analyses were performed in Stata 14.0 (College Station, TX USA).

### Ethical issues

This study was approved by the Ethics Committee in Research of the State University of Rio de Janeiro, protocol CAAE number 64756517.0.0000.5260.

## Results

The prevalence of non-utilisation of dental health services and last dental visit over 12 months ago by Brazilian adults from the State capitals was 1.8% (95% CI 1.6%-2.1%) and 48.1% (95% CI 47.0%-49.1%), respectively. Table 1 presents the individual characteristics according to dental services utilisation outcomes. Most participants were females, had white race/skin colour, had 9–11 years of schooling and individual family income between R$ 501 and R$ 1500.

**Table 1:** Individual predisposing, enabling and need characteristics according to non-utilisation of dental services and last dental visit over 12 months ago.

Variable	Total	Non-utilisation of dental services		Last dental visit > 12 months	
	%	%	95% CI	%	95% CI
***Predisposing demographic***					
Age					
18–21	8.5	2.8	1.9–4.1	40.7	37.5–44.0
22–34	29.2	1.8	1.4–2.4	44.5	42.8–46.3
35–44	19.8	1.9	1.3–2.7	45.3	43.4–47.2
45–64	30.0	1.4	1.1–1.9	49.0	47.1–50.8
≥ 65	12.5	2.0	1.5–2.6	63.5	60.9–65.9
Sex					
Male	45.0	2.4	2.0–2.9	51.8	50.2–53.4
Female	55.0	1.4	1.1–1.7	45.0	43.7–46.3
***Predisposing social***					
Race/skin colour					
White	47.2	1.0	0.7–1.3	43.5	42.0–45.0
Black	10.0	2.8	2.1–3.8	54.9	52.0–57.8
Yellow	1.5	1.1	0.3–4.0	39.3	31.0–48.3
Brown	40.8	2.6	2.1–3.0	52.0	50.5–53.5
Indigenous	0.5	7.9	3.2–18.0	47.8	37.4–58.4
Years of schooling					
0–4	15.8	4.9	4.1–5.9	64.9	62.7–66.9
5–8	19.2	2.4	1.9–3.0	60.7	58.7–62.7
9–11	38.1	1.4	1.1–1.9	46.4	44.9–48.0
> 11	26.9	0.2	0.1–0.3	31.5	29.8–33.2
Social network					
I (Low)	30.5	2.9	2.4–3.5	55.5	53.7–57.2
II	34.7	1.7	1.3–2.1	48.6	47.0–50.3
III	26.6	1.3	0.9–1.8	42.3	40.1–44.2
IV (High)	8.2	0.4	0.2–0.6	36.6	33.9–39.4
***Enabling financing***					
Income (R$)					
≤500	7.1	4.2	3.2–5.5	54.2	50.8–57.6
501 a 1500	43.6	2.3	1.9–2.9	54.5	53.1–55.8
1501 a 2500	12.1	0.8	0.5–1.2	45.3	42.8–47.9
>2500	20.1	0.1	0.1–0.4	32.9	30.9–35.0
Not available	17.1	2.3	1.7–3.0	49.1	46.8–51.4
Health insurance					
No	57.4	3.0	2.6–3.5	56.9	55.6–58.1
Yes	42.6	0.2	0.1–0.3	36.2	34.8–37.7
***Enabling organisation***					
Registered in FHT					
No	38.3	1.9	1.5–2.4	51.2	49.7–52.7
Yes	50.0	1.7	1.4–2.1	46.0	44.5–47.6
Unknown	11.7	2.2	1.6–3.1	46.6	43.9–49.3
***Need perceived***					
Perceived dental health					
Very good / good	70.6	1.5	1.2–1.8	43.2	42.1–44.5
Regular	24.6	2.3	1.8–2.8	58.4	56.6–60.3
Poor / very poor	4.8	5.0	3.6–6.9	65.9	61.4–70.1
Eating difficulties due to oral problems					
No	92.1	1.6	1.4–1.9	47.2	46.1–48.3
Yes	7.9	4.1	3.1–5.5	58.3	55.3–61.1
***Need evaluated***					
Missing teeth					
No	33.2	2.1	1.7–2.7	43.0	41.3–44.8
One or more	58.6	1.5	1.3–1.9	46.4	45.1–47.7
All	8.2	2.8	2.0–3.8	80.6	78.0–82.9

Factor analysis evidenced four factors that explained 89% of the total variance. Table 2 presents the items loadings after oblique rotation. Bartlett's test for sphericity was statistically significant (p<0.001). The model adequacy analysis using the Kaiser-Meyer-Olkin statistic was 0.612. The contextual factors were named as following: 1. contextual predisposing; 2. contextual enabling; 3. contextual perceived need; 4. contextual evaluated need.

**Table 2:** Rotated component matrix presenting factor loadings for the contextual variables included in the factor analysis at city-level.

Variables	Component				Unexplained
	Predisposing	Enabling	Perceived need	Evaluated need	
HDI—income	0.947				0.088
HDI—life expectancy	0.897				0.125
HDI—education	0.807				0.281
Percentage of extremely poor	-0.972				0.067
Percentage of vulnerable to poverty	-0.978				0.047
*Per capita* expenditure in primary care		0.944			0.093
*Per capita* expenditure in oral programs		0.956			0.078
Family oral health teams coverage		0.976			0.054
Oral Impacts on Daily Performances			0.914		0.122
Dental pain			0.940		0.116
DFMT index				0.876	0.175
Need for denture				0.707	0.362

HDI: Human Development Index

Rotation method was oblique with Kaiser normalisation.

In the unadjusted analyses, the predisposing contextual factor was associated with non-utilisation of dental health services and last dental visit over 12 months ago. The enabling contextual factor was also associated with non-utilisation of dental health services. In addition, all individual predisposing, individual enabling (except enabling financing) and individual need variables were associated with both outcomes (Table 3).

**Table 3:** Bivariate analyses between independent variables and non-utilisation of dental services and last dental visit over 12 months ago, Brazil, State capitals, 2013.

Variables	Non-utilisation			Last dental visit > 12 months		
	OR	95% CI	p-value	OR	95% CI	p-value
**Contextual (city-level)**						
Predisposing (greater scores indicate better conditions)	0.63	0.52–0.76	<0.001	0.87	0.77–0.99	0.029
Enabling (greater scores indicate better conditions)	0.82	0.71–0.96	0.011	0.94	0.77–1.14	0.536
Perceived need (greater scores indicate worse perceived oral health)	0.94	0.80–1.11	0.469	0.98	0.93–1.03	0.408
Evaluated need (greater scores indicate worse oral health)	0.86	0.67–1.12	0.266	1.09	0.97–1.23	0.148
**Individual level**						
***Predisposing demographic***						
Age (ref.: 18–21)						
22–34	0.64	0.50–0.84	0.001	1.15	1.04–1.26	0.007
35–44	0.51	0.35–0.74	<0.001	1.22	1.09–1.36	<0.001
45–64	0.64	0.48–0.87	0.004	1.53	1.36–1.73	<0.001
65+	1.07	0.69–1.67	0.759	2.91	2.49–3.39	<0.001
Sex (ref.: female)						
Male	1.67	1.46–1.91	<0.001	1.30	1.24–1.37	<0.001
***Predisposing social***						
Race/skin colour (ref.: white)						
Black	2.71	2.23–3.30	<0.001	1.44	1.31–1.58	<0.001
Yellow	1.01	0.36–2.84	0.983	0.98	0.73–1.30	0.867
Brown	2.05	1.74–2.41	<0.001	1.38	1.28–1.49	<0.001
Indigenous	5.04	2.18–11.68	<0.001	1.10	0.78–1.56	0.596
Years of schooling (ref.: > 11)						
0–4	24.30	13.67–43.20	<0.001	4.79	4.30–5.33	<0.001
5–8	12.04	7.01–20.67	<0.001	3.35	3.02–3.72	<0.001
9–11	5.11	2.92–8.95	<0.001	1.78	1.62–1.96	<0.001
Social network (ref.: IV, High)						
I (Low)	4.20	2.20–8.01	<0.001	2.34	2.06–2.65	<0.001
II	2.53	1.36–4.70	0.003	1.64	1.47–1.84	<0.001
III	2.06	1.13–3.75	0.019	1.27	1.12–1.43	<0.001
***Enabling financing***						
Income (ref.: > R$2500)						
≤ 500	18.02	9.15–35.50	<0.001	2.60	2.34–2.88	<0.001
501 a 1500	10.88	6.23–19.00	<0.001	2.54	2.34–2.76	<0.001
1501 a 2500	4.41	2.39–8.15	<0.001	1.66	1.50–2.20	<0.001
Health insurance (ref.: yes)						
No	8.71	5.77–13.16	<0.001	2.57	2.40–2.75	<0.001
***Enabling organisation***						
Registered in FHT (ref.: yes)						
No	0.96	0.79–1.16	0.643	0.79	0.71–0.89	<0.001
Unknown	0.99	0.72–1.38	0.970	0.81	0.69–0.95	0.011
***Need perceived***						
Perceived dental health (ref.: very good / good)						
Regular	1.46	1.20–1.78	<0.001	1.74	1.63–1.85	<0.001
Poor / very poor	3.52	2.55–4.85	<0.001	2.63	2.31–3.00	<0.001
Eating difficulties due to oral problems (ref.: no)						
Yes	2.24	1.69–2.98	<0.001	1.55	1.41–1.71	<0.001
***Need evaluated***						
Missing teeth (ref.: no)						
One or more	0.62	0.48–0.81	<0.001	1.09	1.02–1.16	0.015
All	1.54	1.13–2.09	0.006	6.55	5.56–7.71	<0.001

In the multilevel analyses, null logistic models were fitted in order to verify the presence of contextual level effects. Null models showed statistical significant random effects for both outcomes, demonstrating there were significant between-city differences. The percentage variance explained by the contextual level was 8.9% for the non-utilisation of dental services and 3.6% for last dental visit over 12 months ago. These proportions are reduced to 2.6% and 1.5% with the inclusion of contextual variables. The average number of observations in each cluster on the second level was 1,000.6, ranging from 520 to 2,568.

The multilevel logistic regression models for the outcome non-utilisation of services are presented in Table 4. Predisposing and enabling contextual variables were inversely associated with non-utilisation of dental services in model 1 and remained associated afterwards. Individual predisposing demographic and social variables in model 2, and individual enabling financing in model 3 were associated with non-utilisation of dental services. In the final model (model 4), adults living in the cities with greater levels of contextual predisposing (OR = 0.77; 95% CI 0.64–0.94) and enabling factors (OR = 0.84; 95% CI 0.75–0.94) had significantly lower odds of non-utilisation of dental services. Sex, race/skin colour, schooling, income, health insurance, perceived oral health and missing teeth remained associated with non-utilisation of dental services.

**Table 4:** Hierarchical multilevel analysis for non-utilisation of dental services, Brazil, State capitals, 2013 (n = 27,015).

Variables	Model 1 OR (95% CI)	Model 2 OR (95% CI)	Model 3 OR (95% CI)	Model 4 OR (95% CI)
**Contextual (city-level)**				
Predisposing (greater scores indicate better conditions)	0.62 (0.52–0.76)***	0.74 (0.61–0.90)**	0.80 (0.65–0.98)*	0.77 (0.64–0.94)*
Enabling (greater scores indicate better conditions)	0.84 (0.76–0.93)**	0.84 (0.76–0.94)**	0.84 (0.75–0.94)**	0.84 (0.75–0.94)**
**Individual level**				
***Predisposing demographic***				
Age (ref.: 18–21)				
22–34		0.75 (0.56–0.99)*	0.80 (0.58–1.10)	1.02 (0.73–1.42)
35–44		0.47 (0.31–0.71)***	0.56 (0.35–0.90)*	0.85 (0.52–1.39)
45–64		0.43 (0.31–0.62)****	0.53 (0.36–0.79)**	0.86 (0.57–1.29)
65+		0.53 (0.34–0.84)**	0.80 (0.48–1.32)	1.18 (0.73–1.92)
Sex (ref.: female)				
Male		1.71 (1.46–2.01)***	1.95 (1.67–2.28)***	1.90 (1.63–2.22)***
***Predisposing social***				
Race/skin colour (ref.: white)				
Black		1.89 (1.60–2.23)***	1.69 (1.44–2.00)***	1.69 (1.43–1.99)***
Yellow		0.96 (0.34–2.72)	0.90 (0.34–2.40)	0.87 (0.34–2.26)
Brown		1.49 (1.27–1.76)***	1.36 (1.15–1.60)***	1.35 (1.14–1.59)***
Indigenous		3.79 (1.72–8.35)**	3.51 (1.60–7.67)**	3.78 (1.68–8.51)**
Years of schooling (ref.: >11)				
0–4		22.26 (13.03–38.01)***	9.75 (6.05–15.71)***	11.51 (7.06–18.76)***
5–8		10.10 (6.10–16.71)***	4.84 (3.10–7.56)***	5.97 (3.78–9.41)***
9–11		4.24 (2.49–7.22)***	2.46 (1.50–4.04)***	2.77 (1.69–4.56)***
Social network (ref.: IV, High)				
I (Low)		2.40 (1.26–4.56)**	2.01 (1.06–3.82)*	1.86 (1.00–3.46)
II		1.69 (0.91–3.16)	1.47 (0.79–2.74)	1.44 (0.79–2.62)
III		1.66 (0.89–3.08)	1.51 (0.81–2.82)	1.52 (0.84–2.76)
***Enabling financing***				
Income (ref.: > R$2500)				
≤ 500			4.52 (2.20–9.27)***	4.45 (2.20–9.03)***
501 a 1500			2.74 (1.54–4.89)**	2.71 (1.54–4.76)**
1501 a 2500			1.87 (0.98–3.58)	1.92 (1.00–3.68)*
Health insurance (ref.: yes)				
No			3.38 (2.21–5.19)***	3.36 (2.19–5.15)***
***Need perceived***				
Perceived dental health (ref.: very good / good)				
Regular				1.10 (0.90–1.33)
Poor / very poor				2.02 (1.49–2.73)***
Eating difficulties due to oral problems (ref.: no)				
Yes				1.32 (0.96–1.81)
***Need evaluated***				
Missing teeth (ref.: no)				
One or more				0.35 (0.26–0.48)***
All				0.47 (0.35–0.63)***
Variance at city level (SE)†	0.091 (0.047)	0.098 (0.051)	0.099 (0.054)	0.095 (0.054)
VPC	0.027	0.029	0.029	0.028

Null model: fixed effect, exponentiated intercept = 0.018 (0.014–0.023); random effect, variance (SE) = 0.320 (0.127), p = 0.011, VPC = 0.089

*P < 0.05

** *P* < 0.01

*** *P* < 0.001

† obtained through random effects

VPC, variance partition coefficient

Table 5 presents the multilevel logistic regression analysis of the relationship between independent variables and last dental visit over 12 months ago. Contextual predisposing variable was associated with the outcome only in model 1. Individual predisposing and individual enabling variables were inserted in models 2 and 3, respectively. In the final model (model 4), the odds of visiting a dentist over 12 months ago was significantly higher for older adults, male sex, brown race/skin colour, low schooling, low social networks, low income, absence of health insurance, poor perceived dental needs and higher number of missing teeth (Table 5).

**Table 5:** Hierarchical multilevel analysis for last dental visit over 12 months ago, Brazil, State capitals, 2013 (n = 26,480).

Variables	Model 1 OR (95% CI)	Model 2 OR (95% CI)	Model 3 OR (95% CI)	Model 4 OR (95% CI)
**Contextual (city-level)**				
1 Predisposing (greater scores indicate better conditions)	0.87 (0.77–0.99)*	0.95 (0.89–1.03)	0.99 (0.91–1.07)	0.99 (0.92–1.07)
**Individual level**				
***Predisposing demographic***				
Age (ref.: 18–21)				
22–34		1.31 (1.18–1.46)***	1.39 (1.25–1.54)***	1.37 (1.23–1.52)***
35–44		1.32 (1.18–1.48)***	1.50 (1.35–1.68)***	1.46 (1.31–1.64)***
45–64		1.47 (1.29–1.68)***	1.79 (1.56–2.06)***	1.72 (1.48–1.99)***
65+		2.39 (2.06–2.77)***	3.26 (2.75–3.86)***	3.17 (2.67–3.77)***
Sex (ref.: female)				
Male		1.42 (1.34–1.51)***	1.52 (1.43–1.61)***	1.50 (1.41–1.59)***
***Predisposing social***				
Race/skin colour (ref.: white)				
Black		1.18 (1.08–1.30)**	1.09 (0.99–1.20)	1.07 (0.98–1.18)
Yellow		0.95 (0.72–1.24)	0.90 (0.71–1.15)	0.88 (0.69–1.13)
Brown		1.19 (1.11–1.28)***	1.11 (1.03–1.19)**	1.10 (1.03–1.18)**
Indigenous		0.94 (0.68–1.30)	0.86 (0.62–1.18)	0.83 (0.61–1.14)
Years of schooling (ref.: >11)				
0–4		3.70 (3.26–4.19)***	2.25 (2.00–2.52)***	2.14 (1.91–2.40)***
5–8		2.90 (2.60–3.23)***	1.87 (1.70–2.07)***	1.80 (1.64–1.98)***
9–11		1.71 (1.55–1.88)***	1.27 (1.16–1.38)***	1.24 (1.14–1.36)***
Social network (ref.: IV, High)				
I (Low)		1.83 (1.61–2.07)***	1.60 (1.41–1.81)***	1.59 (1.40–1.81)***
II		1.39 (1.24–1.57)***	1.25 (1.11–1.41)***	1.25 (1.11–1.41)***
III		1.19 (1.05–1.36)**	1.11 (0.98–1.27)	1.11 (0.97–1.27)
***Enabling financing***				
Income (ref.: > R$2500)				
≤ 500			1.61 (1.43–1.82)***	1.55 (1.37–1.75)***
501 a 1500			1.51 (1.39–1.64)***	1.49 (1.37–1.62)***
1501 a 2500			1.26 (1.13–1.40)***	1.25 (1.13–1.39)***
Health insurance (ref.: yes)				
No			1.81 (1.70–1.92)***	1.76 (1.65–1.87)***
***Enabling organisation***				
Registered in FHT (ref.: yes)				
No			1.01 (0.93–1.10)	
Unknown			1.04 (0.90–1.20)	
***Need perceived***				
Perceived dental health (ref.: very good / good)				
Regular				1.36 (1.27–1.47)***
Poor / very poor				1.76 (1.52–2.05)***
Eating difficulties due to oral problems (ref.: no)				
Yes				0.88 (0.79–0.97)*
***Need evaluated***				
Missing teeth (ref.: no)				
One or more				0.75 (0.70–0.80)***
All				2.85 (2.44–3.34)***
Variance at city level (SE)†	0.050 (0.020)*	0.069 (0.020)***	0.062 (0.019)**	0.063 (0.018)***
VPC	0.015	0.021	0.018	0.019

Null model: fixed effect, exponentiated intercept = 0.963 (0.863–1.074); random effect, variance (SE) = 0.123 (0.030), p<0.001, VPC = 0.036

*P < 0.05

** P < 0.01

*** P < 0.001

† obtained through random effects

VPC, variance partition coefficient

## Discussion

This study showed the influence of contextual characteristics of social vulnerability and health services organisation in the non-utilisation of dental health services in Brazilian adults. However, these associations were not identified for last dental visit over 12 months ago. Individual predisposing, enabling and the need characteristics were associated with both outcomes of dental services utilisation. Sex, race/skin colour, schooling, income, health insurance, perceived dental health and tooth loss were associated with both outcomes. Age, social network and limitation due to oral problems were only associated with utilisation of dental services over 12 months ago.

Our findings suggest the role of the broader social determinants as barriers to access to dental services for the population even when individual social vulnerability conditions are considered [24]. On the other hand, individual socioeconomic characteristics, including schooling and income, which are byproducts of the social context where people live were meaningful determinants of both dental services outcomes [52]. Previous studies reported that people with high oral health needs were also concentrated in the cities with poor contextual social indicators [13, 15], which suggests an inverse care law–individuals with greater oral health needs are those who face greater difficulties in accessing services.

Similar to previous studies, adults who reported poor oral health showed greater difficulties in the dental services utilisation [26, 29, 31]. With regards to tooth loss, the relationship with dental services utilisation was ambiguous. The odd of non-utilisation of dental services was higher for individuals with no missing teeth. This inverse finding is probably due to the contact with dental services for tooth extraction. Thus, the difficulties in accessing dental services concerning tooth loss are better expressed in the other outcome, since edentulous people were more likely to have visited a dentist over 12 months ago, as expected.

Recent utilisation of dental services was inversely associated with higher age. The literature suggests the decrease in the regular utilisation of services with increasing age [22, 26]. Although our findings may be partly attributed to a cohort and period effects, there is a need to implement effective actions aiming to improve access to dental care among older adults [53]. The difficulties imposed by aging, the decrease in the perception of need due to edentulism and the low availability of public service might make this situation even worse [8]. Behavioural and occupational factors have been suggested to explain why male adults use less the health services [54]. However, some studies have demonstrated that gender differences occur in the intermediate age groups, but not among the youngest [27, 28].

Comparing with other countries [55, 56], few studies in Brazil have reported the association between race/skin colour and utilisation of dental health services. Populational and telephone surveys revealed that adults with white race/skin colour had increased access to dental services than other ethnic groups [7] and the disadvantage of health services utilisation among black and brown comparing with white race/skin colour [10]. This study showed that individuals with black, brown or indigenous race/skin colour had more difficulties in accessing dental services. These findings remained significant after adjustment for schooling and income, which in turn corroborate for the role of racial inequities in the access and utilisation of health services, as previously suggested [10]. Besides access to goods and services, racism is also related to health inequities [57, 58].

The association of education and income with dental services utilisation found in the study has been reported in the literature. Besides, a clear social gradient was observed between lower education and poorer utilisation of dental services [8, 12, 28, 29]. The variable health insurance was not able to discriminate those with and without specific dental coverage, and may be a more general proxy of socioeconomic status. Since it is expected that not all participants that hold health insurance have also dental services coverage, the conditions that led individuals to have the insurance also induce them to use dental health services more easily and with more regularity.

Individuals with small social network were more likely to have a last dental visit over 12 months ago. Although it is not expected that social networks eliminate the different types of barriers related to dental services utilisation, social ties could provide a more frequent contact among those who use the health services. Social connectedness and participation in social groups provide material and emotional support that favours the adoption of healthy behaviours, including frequency of dental visits [59–61]. The effect of social networks on dental services utilisation, however, was limited compared to other social determinants [59].

Despite the effect of the contextual characteristics that reflect the organisation of the oral health services on the utilisation of dental services, associations with the individual variable relating to the enrolment in FHT were not found. In the bivariate analysis, the latter was only associated with recent utilisation, which did not remain in the multivariate analysis, differently from that observed in another study [7]. As already stated, the development of oral health policies in Brazil led to an increase in access to services [1, 5]. Therefore, the assessed items of service organisation in the State capitals seem to be important to deal with the social barriers that preclude the utilisation of dental services, although they had no effect on the regular use of dental services. The low presence of oral health teams in the FHT of large population municipalities may have contributed to this result at the individual level [62]. An ecological study involving 63 countries of all six WHO regions also found no correlation between total health expenditure and the utilisation of dental services in the last 12 months, although it found association of the outcome with dentist-to-population ratio [24], as also described in a multilevel approach [20].

The present study did not assess whether the dental services outcomes were related to public or private health services since our objective was to evaluate the general barriers of dental service access. Another relevant aspect is that the information available from the Brazilian National Health Survey was related to the last dental visit and not to the type of dental service regularly used. Previous studies comparing private and public services have shown that the latter is more sought by individuals with lower schooling, low income and poor oral health conditions [21, 63, 64]. Better infrastructure of oral health services was also associated with greater utilisation of public health services [64]. Countries with public health systems, even if partial coverage, tend to present lower socioeconomic inequalities related to the utilisation of dental health services [65].

The cross-sectional nature of this study suggests that any causal inferences must be made with caution [50]. Despite the efforts of standardisation and training, information bias might have occurred due to the large team of interviewers participating in the field work data collection of the survey [44]. On the other hand, the large sample size and its representativeness is a strength of the study. The high number of participants in each city also counterpoises for the low number of groups in the second level (<30) for multilevel logistic regression models [66]. The studied sample included adults from the State capitals. Although this approach allowed the analysis of contextual variables in a multilevel model, the findings might not be generalised to other population groups. In addition, this resulted in a high homogeneity that may explain the low percentage variance explained by the contextual level. Nevertheless, it allowed adequate interpretation and greater applicability of the findings, providing relevant information to support the development of intersectoral public policies that can impact oral health and reduce inequalities in the access and utilisation of dental services.

## Conclusions

Interventions tackling contextual and individual characteristics can reduce barriers to dental services utilisation and therefore contribute to the improvement of the oral health status of the population, mainly among those individuals with the most need for dental services in the Brazilian State capitals. Therefore, it is paramount to develop new strategies and to reinforce current actions for the active inclusion of groups with less access to dental services, including elderly people, males, those with black, brown and indigenous race/skin colour, as well as individuals with low schooling, low income and edentulous people. The implementation of evidence-based intersectoral actions along with those specific to the health sector can provide a more rational and effective use of available resources.

## Abbreviations

SUS: Brazilian Unified Health System
FHT: family health teams
NHS: national health survey
IBGE: Brazilian Institute of Geography and Statistics
PSU: primary sampling unity
VPC: variance partition coefficient
